# Finding the Ion in the RNA-Stack: Can Computational Models Accurately Predict Key Functional Elements in Large Macromolecular Complexes?

**DOI:** 10.1021/acs.jcim.1c00572

**Published:** 2021-06-16

**Authors:** Marco Marcia, Jacopo Manigrasso, Marco De Vivo

**Affiliations:** † European Molecular Biology Laboratory (EMBL) Grenoble, 71 Avenue des Martyrs, Grenoble 38042, France; ‡ Laboratory of Molecular Modelling & Drug Discovery, Istituto Italiano di Tecnologia, Via Morego 30, 16163 Genoa, Italy

## Abstract

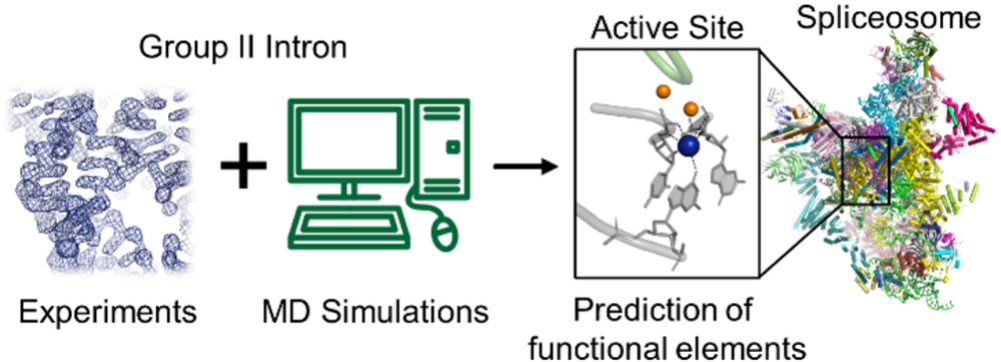

This viewpoint discusses the predictive power and impact of computational analyses and simulations to gain prospective, experimentally supported mechanistic insights into complex biological systems. Remarkably, two newly resolved cryoEM structures have confirmed the previous, and independent, prediction of the precise localization and dynamics of key catalytic ions in megadalton-large spliceosomal complexes. This outstanding outcome endorses a prominent synergy of computational and experimental methods in the prospective exploration of such large multicomponent biosystems.

The challenge of computationally predicting and refining the 3D structure of biological macromolecules has been highly appealing over the last decades. Significantly, such activity can accelerate impactful discoveries in life, environmental, and pharmacological sciences. As a matter of fact, structure predictions by new artificial intelligence-driven algorithms have now achieved unprecedented accuracy, at least for single-subunit proteins. However, predicting 3D structures is often not sufficient to provide mechanistic insights for dynamic biological systems. Computational tools like molecular dynamics (MD) simulations are therefore powerfully used to interpret experimental data and generate integrative models of biological structures or investigate their complex function, dynamics, and even chemical reactions.^1,2^

On the other hand, the power of prospective mechanistic insights from computational studies is still often underestimated. Indeed, it is particularly challenging to detail the functional mechanism of very large macromolecular complexes, such as transcriptional, translational, splicing, or protein/RNA degradation machineries. At a time when technological advances make these multisubunit protein and RNA–protein complexes experimentally tractable, reliably predicting their structures and dynamics can be crucial in rationally guiding and accelerating their characterization and, ultimately, their modulation. In this context, what is the best approach for reliable mechanistic predictions into such large macromolecular systems? How to generate such predictions, and ensure they are valued and exploited by experimentalists? Here, we address these questions with a recent example that shows how the integration of computational and experimental data helped provide key predictive structural and mechanistic insights into vital, ubiquitous, and medically relevant splicing machineries.

Splicing is a two-step biological reaction whereby introns are excised from precursor RNA molecules and exons are ligated into mature functional protein-coding or noncoding transcripts. In detail, splicing chemistry consists of two sequential scissions of phosphodiester bonds at the 5′- and 3′-intron/exon junctions, respectively. Both reactions, which are S_N_2-like nucleophilic additions, occur within an active site comprising two divalent metal ions that coordinate and activate the reacting residues (Figure 1A).^3,4^ This two-metal-ion reaction chemistry is identical to that of other nucleic-acid-processing protein enzymes, such as endo/exonucleases and polymerases. All these complex enzymes catalyze the scission or synthesis of phosphodiester bonds in DNA/RNA, respectively. The ubiquitous nature of such metal-aided structural architecture of the catalytic site is corroborated by the large therapeutic spectrum of drugs that target two-metal-ion enzymes and are thus broadly used to treat cancers and viral infections.^5^

**Figure 1:**
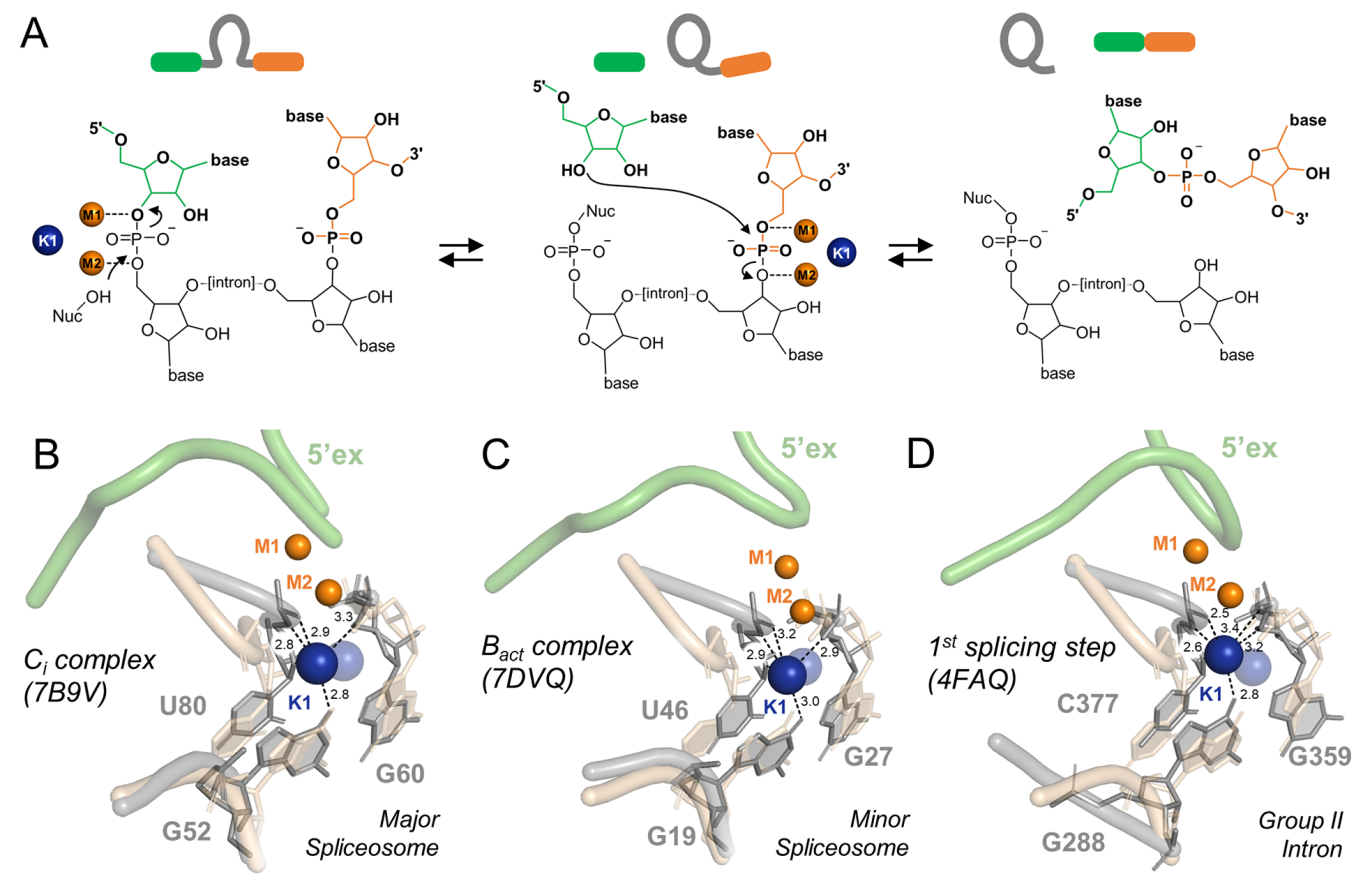
K1 is required for both steps of forward splicing. (**A**) Schematic representation of the two steps of the splicing reactions. The 5′- and the 3′-exon are highlighted in green and orange, respectively. Catalytic ions M1–M2 (orange) and K1 (blue) are shown as spheres. Black arrows indicate nucleophilic attacks, while “Nuc” indicates the reaction nucleophile. (**B**–**D**) Structural superposition of the K1 binding model over the major spliceosome (C_i_ complex, panel **B**), the minor spliceosome (B_act_ complex, panel **C**), and the group II intron (panel **D**). Catalytic ions M1–M2 (orange) and K1 (blue) are shown as spheres. The nucleotides coordinating K1 (gray) are represented as sticks, while the 5′-exon (green) is shown as a cartoon. The predicted K1 binding model (beige) is depicted in semitransparent representation. The predicted K1 ion (blue) is depicted as a semitransparent blue sphere. Black dotted lines represent K1 coordination distances in angstrom.

In eukaryotes, splicing is mostly catalyzed by megadalton-large ribonucleoproteins called the spliceosomes, which exist in two isoforms: the major spliceosome, which processes 99% of all transcripts in humans, is formed by the U1/U2/U3/U4/U5/U6 small nuclear ribonucleoproteins (snRNPs): and the minor spliceosome, which processes the remaining 1% of human transcripts, is formed by the U11/U12/U4atac/U5/U6atac snRNPs.^6^

Until recently, mechanistic insights into spliceosomal complexes were mainly based on phylogenetic analysis, chemical-mapping, and enzymatic and biochemical assays.^7^ Limitations in obtaining more detailed mechanistic insights were primarily due to the large dimensions, heterogeneous biopolymeric composition (6 large RNAs and hundreds of protein subunits), and dynamic assembly of the spliceosome along the catalytic cycle. In fact, high-resolution structural insights have been initially obtained only indirectly from crystallographic work on the so-called group II self-splicing introns,^4,8−10^ which are the evolutionary ancestors of the spliceosomes. Nonetheless, with 38 new cryoEM 3D structures produced in the last five years, we have now reached an increasingly good understanding of the dynamic assembly and remodeling of spliceosomal protein and RNA subunits throughout the splicing cycle.^7^ Despite this progress, so far, the resolution of the available structures (>3.0 Å, most structures at >4.0 Å) had remained a limiting factor in defining the catalytic site’s atomic details.

Among other properties, the dependence of the spliceosome on potassium ions, which had been functionally reported already since the early 1980s,^11^ remained unexplained at the molecular level. A nearly 40-year-long research effort to explain this enzymatic observation was crucially informed and guided by structure–function studies on the group II introns and by closely related computational analyses of various classes of nucleic-acid-processing protein and RNA enzymes (see below). These analyses had led to the prediction that a specific potassium ion (named K1) could be localized near the catalytic site of the spliceosome and thus contribute to catalysis through precise structural and functional interactions (Figure 2).^12^

**Figure 2:**
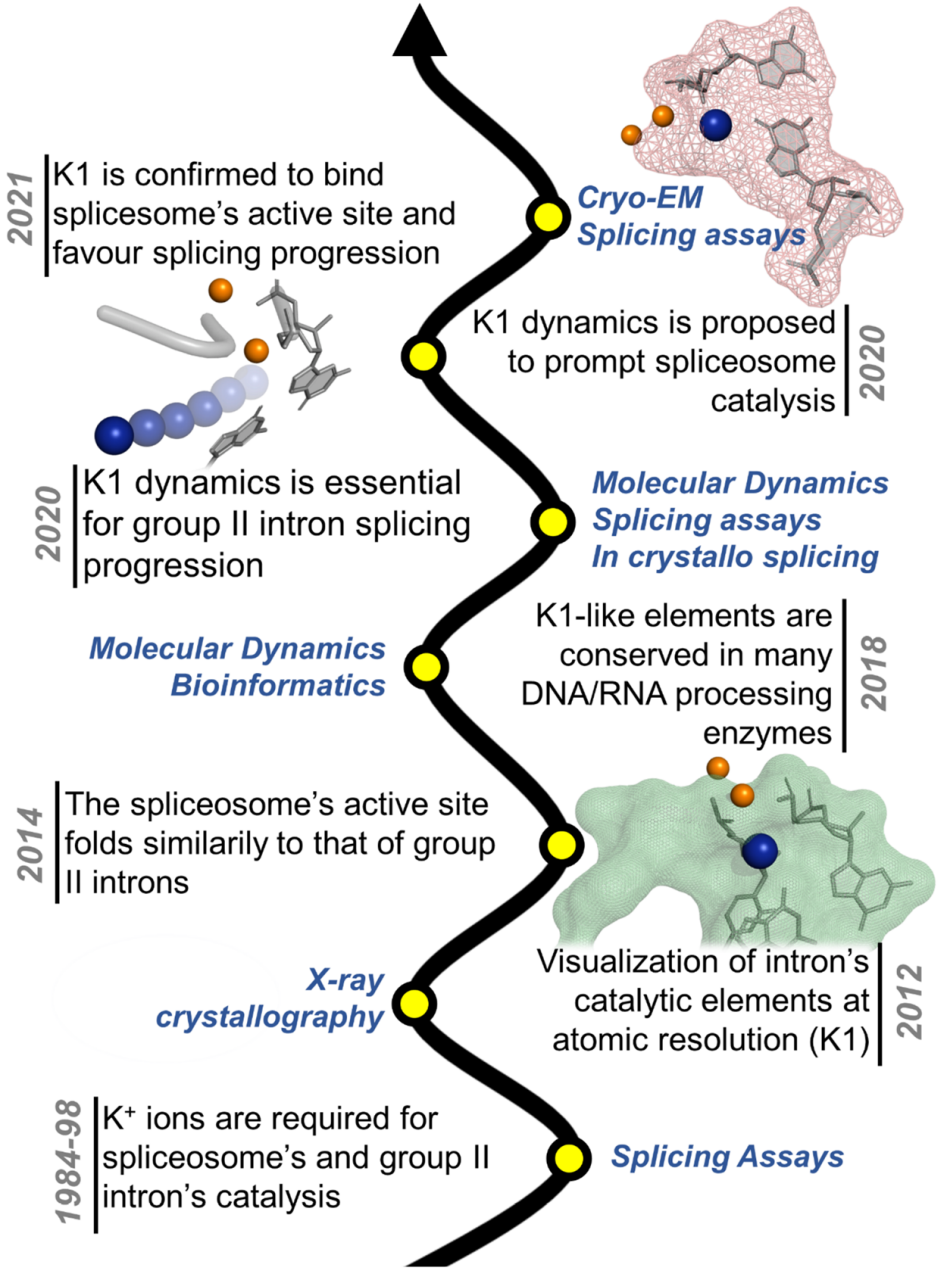
Computational and experimental milestones that marked the progressive discovery of the role of K1. Functional studies initially revealed the importance of potassium for group II intron and spliceosomal splicing,^11^ but only three decades later was the K1 potassium ion identified in intron active site by crystallography.^8^ Subsequent generalization of the K1 importance in multiple classes of nucleic-acid processing enzymes by means of structural/computational analyses,^12^ and the elucidation of the functional role and dynamics of K1 by structural, enzymatic, and MD analyses^18^ led to the prediction that a similar ion would also bind within the spliceosomal active site. This ion and its dynamics have now been successfully identified in the major and minor spliceosomes.^16,17^

K1 was first identified in the active site of the group IIC intron from the bacterium *Oceanobacillus iheyensis* in 2012 by crystallizing this ribozyme in the presence of different mono- and divalent metal ions and by performing anomalous diffraction X-ray studies.^8,13−15^ In the *O. iheyensis* intron structures, one of which was solved at 2.7 Å resolution, K1 is coordinated by active site residues G288, G359, and C377 (Figure 1**D**).^4,8,13^ At that time, it was questioned whether K1 was a conserved active site element or idiosyncratic only to the *O. iheyensis* intron, and as a consequence this ion was not modeled in other lower-resolution structures of homologous group II introns.^10^

Subsequent systematic integration of evolutionary and structural alignments, electrostatic potential calculations, and MD simulations made it possible to appreciate the ubiquitous presence of positively charged residues surrounding the active sites of several and diverse nucleic-acid-processing enzymes.^12^ These basic residues were structurally and functionally analogous to the group II intron K1, suggesting evident mechanistic similarities in enzymes where K1, or K1-like residues, were likely to provide a key functional contribution for nucleic acid processing. Indeed, in these enzymes, K1-like residues act in synergy with the previously recognized two-divalent-metal-ion core and specifically contribute to shape the electrostatics of the active site, modulating the orientation and dynamics of key reacting residues for catalysis. For instance, microsecond-long equilibrium MD simulations have shown that the absence of K1-like residues in polymerase-η induces a distortion in the reaction substrates and disrupts the Michaelis–Menten complex, thus hampering catalysis.^12^ Such comparative analysis of group II introns, exo/endonucleases, and polymerases offered solid and accurate bases to predict the presence, identity, and exact location of K1 also in the spliceosome (Figure 1**B**–**D**).^12^ In more detail, through our analysis and simulations, we predicted K1 to be located at a site coordinated by G52, G60, and U80, which are the evolutionarily and structurally homologous residues to the group II intron G288, G359, and C377, in the structure of the major spliceosomal C complex. At *that* time, this was the most reliable structure to model the location of K1 (PDB ID: 5LJ3; Figure 1**B**–**D**).

Remarkably, in the last few weeks, two new structures below 3.0 Å resolution of the major spliceosomal C_i_ complex^16^ and the minor spliceosomal B_act_ complex^17^ provided the necessary information to experimentally identify and localize specific structural and functional elements in and around the spliceosomal active site. Outstandingly, K1 was identified in the exact same position as predicted by our analyses and simulations, back in 2018 (Figure 1**B–D**).^12^ In the major spliceosome, K1 is coordinated by G52, G60, and U80 (U6 snRNA) (Figure 1**B**), and in the minor spliceosome, K1 is coordinated by G19, G27, and U46 (U6atac snRNA) (Figure 1**C**—see also coordination distances). The overlap of our predictive model with these two new cryoEM structures returned an RMSD of ∼0.6 Å (Figure 2), calculated using the first shell coordination of K1—which is the exact K1 coordination shell we had predicted.^12^ In both structures, K1 appears, indeed, crucial for catalysis and specifically engaged both in the first (in the minor spliceosome B_act_) and the second (in the major spliceosome C_i_) steps of splicing.^16,17^ The functional engagement of K1 was also confirmed by splicing assays, monitored using a stalled major spliceosomal C complex.^16^

But K1 is not just a static structural component of the intron and spliceosomal active site. Extensive computational studies performed since 2012 on group II intron X-ray structures at multiple stages of catalysis had contributed to explaining the complex functional role and dynamics of K1 throughout the splicing cycle.^18^ Indeed, multimicrosecond equilibrium MD simulations and free-energy calculations (metadynamics) have shown that K1 is dynamically bound and released to and from the intron active site favoring functionally important conformational rearrangements of the intron’s active site, which are required for exchanging the reaction products and substrates in between the first and second steps of splicing.^18^ Notably, according to our results, this sequence of events is directly triggered by the protonation of one catalytic residue just after the first splicing reaction, as indicated by quantum-mechanics/molecular-mechanics (QM/MM) simulations coupled with enzymatic assays, mutagenesis, and structural characterization.^18^

Importantly, the K1 dynamics observed in the intron are analogous to dynamics of K1-like residues in protein enzymes. In polymerase-η, the altered dynamics of K1-like second-shell basic residues was shown, via equilibrium MD and enhanced sampling simulations, to disfavor the formation of the Michaelis–Menten complex, ultimately impairing the catalysis.^12,19^ Similar positively charged second-shell residues have been recently shown via MD simulations to play an active role for catalysis in other nucleic-acid processing metalloenzymes, as in the case of λ-exonuclease, dUTPases, and human exonuclease 1 enzymes.^20−22^ As a result of these studies, and in light of the extended similarities between the intron and protein enzymes, it seemed likely that similar dynamic events are ubiquitous and necessary for nucleic acid processing and would thus also occur in the spliceosome.^18^ Remarkably, by comparing the new spliceosomal cryoEM structures with previous ones obtained at various steps throughout the splicing cycle, K1 appears to be indeed dynamic and transient, i.e., bound to the active site for the catalytic steps but released during conformational rearrangements.^16^

Taken together, these results illustrate how comparative structural, functional, and evolutionary studies on the group II intron coupled with extensive molecular simulations and free energy calculations of both such a challenging system and other convergently evolved nucleic-acid-processing enzymes enabled accurate predictions into the intricate catalytic core of the spliceosomes. Notably, experimental characterization of these complex systems has inevitably lagged behind due to the complexity of these megadalton-large ribonucleoproteins.

The recent experimental confirmation of the accuracy of such structural and mechanistic predictions offers increased confidence in the predictive power of such computational simulations, when appropriately integrated with evolutionary analysis and experimental data. Predicting such precise structure/functional correlations between the catalytic heteronuclear metal clusters of the group II intron and the spliceosomes can guide the mechanistic interpretation of high-resolution structural data, facilitating the functional dissection of these vital splicing machineries. This example thus shows that a computationally informed approach can deliver valuable insights into complex protein-nucleic acid systems, particularly those relevant to human diseases.

This is a time when RNA-targeted drug development is emerging as a viable strategy for developing new therapeutics, including against the spliceosome itself, as shown by the recent FDA approval of risdiplam for the treatment of spinal muscular atrophy.^23^ The precise modeling and design of organic and inorganic compounds, located at binding pockets in and around catalytic centers of complex ribonucleoprotein machineries, can be of great value for fostering structure-based drug design even before high-resolution structures are experimentally determined. In this scenario, the confirmed accuracy of mechanistic predictions based on integrated MD simulations and structural data, about the functional role of K1,^12^ reinforces the notion that positively charged second-shell residues are indeed essential regulators of nucleic-acid-processing chemistry together with the previously recognized two divalent metal ions.^12^ This observation provides precise grounds for understanding the mechanism, modulating function, and targeting many medically relevant enzymes beyond splicing complexes with small molecules. For instance, our predictive structure–function insights can rationally support the biotechnological engineering of CRISPR-Cas systems, which are now emerging as potentially powerful “drug machines” for gene or RNA editing and for personalized gene therapies. More broadly, integrated and computationally driven approaches, as the one we discuss here, can serve as a useful reference (and confidence boost, too) for future modeling and prospective mechanistic interpretation of many other large macromolecular machineries that are essential for life and critically involved in diseases but difficult to experimentally characterize at high resolution. Critical examples of such complex machineries include membrane-embedded supercomplexes or ribonucleoproteins formed by highly structured long noncoding RNAs.^24^ Prospective applications of molecular simulations for structure refinement and mechanistic insights will undoubtedly play a prominent role in the incessant exploration of such complex biological multicomponent systems.
